# Novel High Accuracy Resolver Topology for Space Applications

**DOI:** 10.3390/s21144711

**Published:** 2021-07-09

**Authors:** Jon Santiso-Zelaia, Gaizka Ugalde, Fernando Garramiola, Ion Iturbe, Izaskun Sarasola

**Affiliations:** Faculty of Engineering, Mondragon Unibertsitatea, 20500 Arrasate-Mondragon, Spain; gugalde@mondragon.edu (G.U.); fgarramiola@mondragon.edu (F.G.); iiturbeb@mondragon.edu (I.I.); isarasola@mondragon.edu (I.S.)

**Keywords:** resolver, rotary sensor, high accuracy sensors, resolver topologies, wound rotor, variable reluctance, space, electromagnetic sensor

## Abstract

In recent years, the space industry has experienced a significant change mainly due to the incursion of private companies, which has shaken up the sector. This new situation allows for a reduction regarding the reliability of conventional instrumentation for space while reducing the development time and manufacturing volume. Consequently, even though it has been typical to use equipment that was previously tested in space, this could be the right moment to introduce new technologies due to the previously mentioned reasons. One of the interesting technologies with great potential is the rotary sensor in applications with motors. Historically, the resistive potentiometer has been the most used due to its simplicity and robustness; however, it has several drawbacks. Due to this, the aim of this paper is to identify an interesting rotary sensor. Hence, in this article, different sensor types are studied. Then, we review the literature regarding resolvers in order to find the best topology. We designed and compared different single speed absolute position resolvers to find the ones that offered the best results. In this process, a novel resolver topology was designed that improved on the performances of any other studied topology.

## 1. Introduction

In recent years, the space industry has experienced a significant change mainly due to the incursion of private companies, which has shaken up the sector. As an example, the projects Kuiper of Amazon [1] or the Starlink mission of SpaceX [2] can be highlighted, as they plan to put into orbit constellations of thousands of satellites. This new “era” is called New Space [3], and its main characteristics are the focus on volume manufacturing with tighter schedules aiming to reduce the costs.

This new situation leads to new manufacturing processes that allow for reducing the requirements for conventional instrumentation for space applications. This is intended to be compensated for by reduced development times, lower budgets, and increased manufactured units. Hence, even though it was usual to use equipment that was previously tested in flight, due to the previously mentioned reasons, new systems that were not previously validated in flight could be introduced to space applications.

Among these systems, gimbals [4], solar arrays [5], optical equipment [6,7], and antenna pointing systems [8,9] can be found. All of these have in common that they are driven with a mechanism that is considered to have great potential for innovation.

The mechanism can be composed of a motor (stepper, brushless, Limited Angle Torque (LAT) motor...), a speed or position rotary sensor (electric resistive potentiometer or electromagnetic resolver), and a mechanical gear. This work focuses on the sensor, more specifically on rotary sensors as it is considered that they have a great potential for innovation.

As explained in Section 2.1.1, one of the most common sensors used in space applications is the resistive potentiometer. However, in order to ensure a high-performance of the mechanism in its corresponding application, it is necessary to provide the controller with an accurate position reading, and this technology has some intrinsic drawbacks that could be solved by replacing it with another type of sensor.

As a consequence, the aim of this paper is to design and develop a novel sensor topology that fulfills the requirements of the New Space era, summarized next. One of the required characteristics is the simplicity of the device in terms of the geometry/construction in order to speed up the manufacturing and assembly processes. Additionally, even though it has been said that the volume manufacturing and lower budget could compensate the performances of new technologies, there are applications [10] that require encoders with up to 25 bit resolutions (0.04 arcseconds) [11].

Hence, the developed sensor must provide high accuracies for one revolution, i.e., the sensor must provide an accurate absolute position. Moreover, considering the harsh conditions of space (high vibrational loads during launch, wide temperature ranges from −270${}^{\circ }$C to more than 2000 ${}^{\circ }$C [12]), the sensor has to be robust in order to ensure its correct operation.

In Section 2, different rotary sensor technologies are analysed in order to check their advantages and disadvantages. The most appropriate sensor technology is selected—in this case, the resolver. In Section 3, a review of the literature regarding resolvers is carried out. In Section 4, different resolver topologies are designed and simulated, where a novel resolver topology was developed. In Section 5, the obtained results are compared and discussed, and we demonstrate that the novel resolver topology greatly improved the performances shown by other topologies from the literature. Finally, in Section 6, our conclusions are given.

## 2. Rotary Sensor Classification

There are many rotary sensors that could be considered for space applications; hence, in this section, different types will be classified into three main groups, which are the potentiometers, encoders, and electromagnetic sensors. Moreover, among these groups, different types can be found. Additionally, the advantages and disadvantages of each topology are pointed out.

### 2.1. Potentiometers

The potentiometer principle of operation consists of a fixed resistive part and a rotary part working as a voltage divider. The sensor is supplied with a constant input voltage, and, depending on the position of the rotary part, the output voltage varies. Knowing the principle of operation, different potentiometers are found, which are listed as follows.

#### 2.1.1. Resistive Potentiometer

Regarding the resistive potentiometer, the rotary part has a conductive wiper that slides against the fixed resistive part [13]. Due to their vast heritage in aerospace applications and the great knowledge regarding their behaviour, this type of sensor is suitable for sensing the position. These sensors provide accurate and repeatable results, they have a simple physical principle, and they are low cost. However, they have a dead band, and thus they cannot ensure the continuous angle readout over 360${}^{\circ }$, although this problem is resolved by using two potentiometers. Additionally, due to the permanent contact, wear debris is caused, which could affect the performance of the sensor.

Thus, the main advantage, as has been previously said, is that it has a vast heritage in aerospace applications and has been proven to be a robust and tested solution. However, its limited life due to contact and wear debris and the existence of a dead zone make it of interest to find an alternative for this sensor.

#### 2.1.2. Magnetoresistive Potentiometer

In this case, the fixed resistive part consists of a magnetoresistance, with the rotary part magnetically working as a voltage divider as previously explained. Hence, depending on the position of the rotary part, the applied magnetic field on the magnetoresistance changes, which consequently changes the resistance value, and thus the output voltage varies [14].

The advantage of this technology is that, as there is no contact between the rotary and the fixed part, it provides a longer life. However, the sensor is very sensitive to external magnetic fields, and hence it may not be the best option for high accuracy applications.

#### 2.1.3. Membrane Potentiometer

This potentiometer, as well as the previous potentiometers, works as a voltage divider. It consists of several separated layers that create contact between each other after applying pressure by mechanical or magnetic means [15].

This sensor offers very low noise with high linearity output and a longer life than conventional resistive potentiometers. On the contrary, as well as the resistive sensors, these potentiometers have a dead zone, and even though they have a longer life, it is limited, as its working principle is based on contact.

### 2.2. Encoders

The encoder is another type of rotary sensor. It codifies the shaft position in different types of signals (digital binary signal, pulses…), and, depending on the provided signal, it can be classified as incremental or absolute [16].

- Incremental encoder: measures a change in position, providing a pulse per position.
- Absolute encoder: provides an absolute value of the shaft position. This is done by means of providing the required number of bits per position to achieve the needed resolution. These encoders could be classified as:
  - Single-turn encoder: provides the absolute position in each revolution but does not provide the number of revolutions.
  - Multi-turn encoder: apart from the absolute position per revolution, counts the number of revolutions performed.

On the one hand, if the position record is lost with incremental encoders, the shaft must be sent back to a known position to enable the reading. However, it has a simple pattern due to requiring only one bit (two if the quadrature is taken into account), and hence higher resolutions could be achieved. On the other hand, the absolute encoder requires a certain number of bits per position; hence, the achievable resolutions are lower than with those of the incremental. However, the exact position is known every time and thus it does not need repositioning after a position loss.

Apart from the given encoder classification depending on the signal they provide, they can be classified depending on how they codify the shaft position into a electronic signal. These types are optical, magnetic, and capacitive [17].

#### 2.2.1. Optical

The optical encoder consists of three main parts: the light emitter, the light sensor, and the disk placed between the emitter and sensor, which is radially differentiated in transparent and opaque regions, so as to let the light sent by the emitter pass through the disk or block it. The passed light will be detected by the sensor, and a digital signal will be generated to enable the position reading. The advantages of this technology are that it has a very high accuracy and that the electronics for acquiring the measurements are already developed. However, these devices are more difficult to mechanically install, and they are more fragile.

#### 2.2.2. Magnetic

These encoders, as well as the optical ones, consist of three main parts: a disc with multiple poles distributed along its perimeter, the sensor, and a conditioning circuit. The disc generates a variable magnetic field as it rotates. The field is detected by the sensor, which can be a Hall sensor [18,19] or a magneto-resistive device [20,21] (these were developed for space applications), and its output is later modified by the conditioning circuit to generate the desired output. These type of encoders offer lower accuracies than the optical ones. However, they are smaller, cheaper, and more robust to harsh environments [22].

#### 2.2.3. Capacitive

This encoder consists of two patterns of bars or lines, with one set on the fixed element and the other set on the moving element, together forming a variable capacitor configured as a transmitter/receiver pairing [23,24]. As the encoder rotates, an integral Application-Specific Integrated Circuit (ASIC) counts these line changes providing standard quadrature and commutation outputs [25]. Even though the accuracy of this technology could be increased by using an ADC of higher bits without the need of the encoder’s redesign, they typically provide low accuracies.

### 2.3. Electromagnetic Sensors

These sensors are based on a electromagnetic field generation part by means of an electric current (as it could be a wound rotor) and a electromagnetic generator part in which a voltage is induced. In this group the synchro, the resolver and the inductosyn could be found [26], which are discussed next.

#### 2.3.1. Synchro

**Figure 1:**
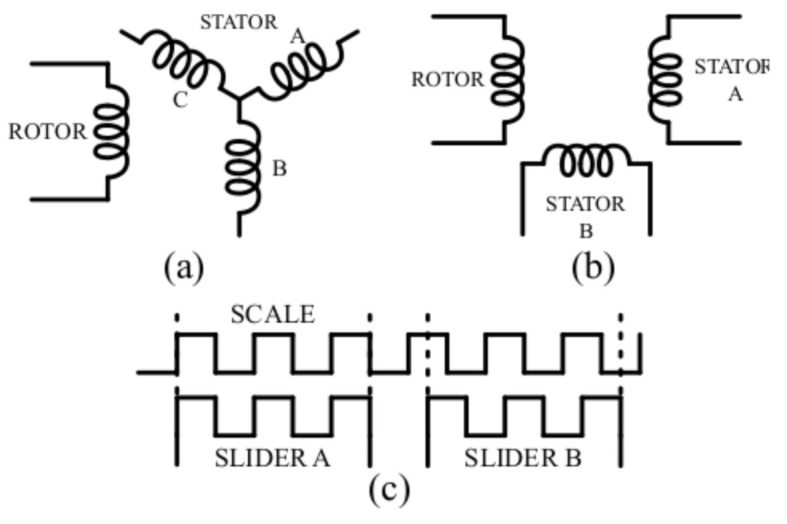
Electromagnetic sensor equivalent circuits: (**a**) synchro, (**b**) resolver, and (**c**) inductosyn.

The sychro is a rotating transformer that consists of a rotor with one or three windings inside a fixed stator with tree windings. Its electrical representation is shown in Figure 1a. The rotor winding is excited with an AC voltage, which induces a voltage in the stator windings proportional to the cosine of the rotor shaft’s position.

These type of technologies, unlike encoders, are a robust solution. They provide an “infinite” life as they are contactless due to their inductive operation principle. However, they offer a low resolution.

#### 2.3.2. Resolver

The resolver, as well as the synchro, is a rotary transformer that converts the shaft angle to an absolute analogue signal, which consists of almost the same structure, in which the windings of the stator are displaced electrically 90${}^{\circ }$ instead of 120${}^{\circ }$ as in the synchro. The electrical representation is shown in Figure 1b. The rotor of the resolver, equal to synchro’s, is excited with an AC voltage that induces a voltage in the stator windings proportional to the cosine of the rotor shaft’s position.

This technology is very robust, and, as it is also based on the inductive operation principle, it has an “infinite” life while providing very high accuracies. However, it is a heavy solution as it is like having an additional motor.

#### 2.3.3. Inductosyn

The inductosyn is generally known as the most accurate angular transducer with accuracies up to 0.5 arcseconds. The rotary inductosyn, as well as the synchro and the resolver, consists of two parts. In this case, these parts are the scale, which corresponds to the rotor, and the slider, which corresponds to the stator. Both the scale and slider have a printed circuit track forming a continuous rectangular waveform. In the case of the slider, it has two different tracks shifted 90${}^{\circ }$ as shown in Figure 1c.

The inductosyn provides a very high accuracy. It has an “infinite” life and is a robust solution. However, this technology requires an external zero for granting the absolute measurement.

### 2.4. Trade Off/Conclusions

As it can be seen, there is a great variety of rotary sensors ready for space applications and some others with great potential. In particular, resistive potentiometers have a vast heritage in aerospace applications due to their robustness and because they are already a tested solution; however, they offer a limited life and have a deadzone, which has to be compensated for with an additional potentiometer. Membrane potentiometers could be a replacement as they last longer, but they still have the same drawbacks as the resistive potentiometers. Another option could be the magnetoresistive potentiometers, which offer “infinite” life even though they are sensitive to external magnetic fields.

The optical and magnetic encoders provide high precision. However, the high precision encoders for space [11] are very expensive due the manufacturer’s dominance of the sector, and thus it could be interesting to find another alternative. The magnetic and capacitive encoders provide lower accuracies and are more sensitive to external interferences, even though the magnetic is more robust.

Electromagnetic sensors, such as the resolver or the inductosyn, have “infinite” life and high accuracies (unlike the synchro, which provides lower accuracies), as it mainly depends on the ADC’s resolution. Additionally, the resolver is a robust solution as its structure is identical to that of a electric machine. On the contrary, due to this structure, this technology is heavier than others.

Considering this and taking into account the requirements of space applications mentioned in Section 1, as a general conclusion, even though any of the above mentioned technologies could be suitable due to their advantages, the resolver appears to be the most attractive technology. This is because it has “infinite” life, could provide very high accuracies (depending on the ADC), is a robust solution, and provides shaft position information even if it remains still. Consequently, its main drawback of weight it is more than compensated.

## 3. Review of Resolvers

As was concluded in the previous section, the resolver is a suitable option as a reliable and accurate position sensor for space applications.

Among the resolvers, after reviewing the literature, two main groups were differentiated based on their physical principle: the Variable Reluctance (VR) resolvers and the Wound Rotor (WR) resolvers. As its name suggests, the position sensing in the VR resolvers is based on the reluctance variation as the air-gap changes. This is done by means of a specially shaped rotor.

The stator is composed by the excitation coil and both the sine and cosine coils. In the case of the WR, as its name suggests, instead of having a excitation coil in the stator as in the VR resolver, it is wound in the rotor. The stator only contains the sine and cosine windings. In this case, in order to supply the rotor’s excitation winding and keep the resolver brushless, a Rotary Transformer (RT) is required. Thus, the conventional brushless WR usually has a RT.

Regarding the performance of the VR resolvers, in [27], two optimization guidelines were given. Then, a two pole VR resolver was simulated and tested, and the obtained maximum error decreased from 6.7${}^{\circ }$ to 0.614${}^{\circ }$ after applying fractional slot sinusoidal windings. In [28], VR resolvers were analysed under different uneven magnetic field conditions. In [29], a ×4 resolver was developed reporting an error of ±0.5${}^{\circ }$. In [30], another multiturn VR resolver was developed with one resolver for absolute position sensing and another for precision—one as a sinusoidal airgap length resolver and the other as a sinusoidal airgap area resolver. In [31], different pole and winding configurations were studied, with an error of 0.4${}^{\circ }$ for the best case resolver.

Axial type VR resolvers were also studied. In [32,33], different speed resolvers (×2, ×4, and ×8) were studied, obtaining an error of 1${}^{\circ }$ for a ×8 resolver. In [34], another axial VR resolver was optimized in order to improve its accuracy, obtaining a maximum error of 0.2${}^{\circ }$. In [35], a disc type rotor axial VR resolver was developed, with an error of 0.25${}^{\circ }$.

Some of the studies were focused on improving the manufacturing process or the structure of the VR resolvers. In [36], a slotless VR resolver was developed obtaining an error of 1.2${}^{\circ }$ in simulation and an error of 2.6${}^{\circ }$ with the prototype. This topology significantly reduced the radial dimensions of the resolver. In [37], a winding configuration where different windings are non-overlapped was developed, simplifying the manufacturing process and obtaining an error of 2${}^{\circ }$ with a ×5 prototype. In [38], the relationship between the number of stator slots, winding polarities, and rotor poles for VR resolvers was investigated. A ×5 prototype showed an achievable error of ±1.4${}^{\circ }$.

In [39], a planar coil VR resolver was developed. In [40], a novel two-pole VR resolver structure was proposed. It consisted of two different resolvers of one pole each, axially separated, shifted 180${}^{\circ }$, and tied to a non ferromagnetic shaft. The obtained error was of 1.35${}^{\circ }$. In [41], a novel resolver structure was proposed where a stator of a rotary transformer was used as the excitation coil, the stator of the WRresolver was kept as typical, and a ferromagnetic plate was used as the rotor.

The other main group of resolvers is composed of the WR resolvers. Regarding these, all of the reviewed articles were axial WR resolvers. In [42], the static eccentricity (one of the most common problems in resolvers) was studied, and a solution was proposed, which was to integrate a semi-damper winding into the resolver. The original error decreased from around 5.5${}^{\circ }$ to 1.1${}^{\circ }$.

In [43,44], fractional slots were used to reduce the harmonic content of the position signals, and different pole/winding combinations were studied in order to find the best fractional slot combination. An error of 0.36${}^{\circ }$ was reported. In [45], the authors attempted to remove the RT integrating the rotor excitation coil in the resolver itself. The performance was compared with a conventional axial flux resolver. The error of the conventional resolver was 4.2${}^{\circ }$, and the error of the proposed resolver without RT was 5.4${}^{\circ }$. In [46], a similar structure was developed.

In [47], four different resolvers—both VR and WR—were studied and compared. The study discussed the challenges of FEM with resolvers. In this study, the validity of the Total 271 Harmonic Distortion (THD) was evaluated as a criterion for accuracy. They reported an error of 0.25${}^{\circ }$ for a five-pole pair VR resolver and an error of 1.2${}^{\circ }$ for a three-pole pair WR resolver. The other two one-pole pair VR resolvers reported an error of 2.3${}^{\circ }$.

The VR resolvers are typically smaller, whereas the WR resolvers are bulkier as they usually require a RT. Additionally, the VR resolvers demonstrated slightly better accuracies than the WR resolvers for the same speed (0.25 deg error with VR vs. 0.36 deg error with WR as seen in Table 1).

However, as has been previously mentioned, this study focused on finding a suitable rotary sensor for New Space. This means that the sensor must fulfil high accuracy requirements while being adequate for volume manufacturing. Taking this into account, the VR resolvers have a more complex rotor structure, and hence they require a more precise manufacturing process, which could delay their supply time. The WR resolvers could be more adequate even though they have demonstrated a slightly worse accuracy.

Consequently, in order to find a suitable WR resolver for New Space, in the next section, different topologies were designed and simulated. A novel topology was developed, demonstrating much better results than those shown in Table 1.

## 4. WR Resolver Design for New Space

In this section, five different WR resolvers are studied. The aim was to compare them in terms of the output signal THD, accuracy, power consumption, and weight in order to identify the best topology. Among these resolvers, three conventional slotted resolvers with different winding configurations, a novel slotless resolver with toroidal winding, and a slotted resolver with a toroidal winding were designed.

The slotted resolvers were selected because they represent the most common WR resolver. The toroidally wound slotless is supposed to be better in terms of accuracy but it was of interest to check its performance taking the other characteristics described later into account. The toroidally wound slotted resolver was selected to combine the main characteristics of both slotted and slotless designs.

**Figure 2:**
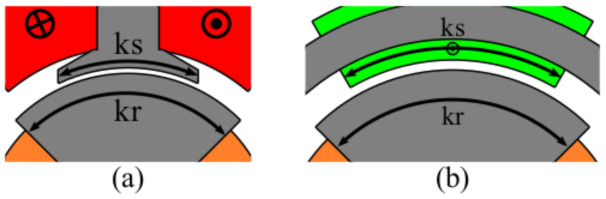
Optimized resolver parameters (**a**) for slotted resolvers and (**b**) for slotless resolvers.

These resolvers were designed as single speed (1×) to provide an accurate absolute position. Each resolver was simulated and optimized by means of Finite Element 302 Method (FEM) simulations carried out with Altair Flux™. Based on the authors’ previous experience on designing a WR resolver for space, in this study, only two geometric parameters—kr & ks—were selected for the optimization purpose, as shown in Figure 2. On the one side, the rotor parameter kr, is common for every resolver and defines the span of the rotor yoke next to the airgap. On the other side, the stator parameter ks defines the stator teeth span for the slotted ones and the winding span for the slotless ones.

For each kr and ks, the obtained EMFs were studied in order to find the combination with the least THD. Additionally, for each parameter combination, different skew values were applied and simulated to eliminate the existent lower frequency harmonics and, hence, improve the THD. Consequently, the best parameter combination and the best skew value were selected for each topology. The parameter combination offering the best EMF without skew and with skew may not be the same as demonstrated in the next sections.

Next, the best EMFs of each resolver were compared with an ideal sine and cosine, and then they were converted to the angular position. The position reading was carried out in MATLAB by means of a Resolver to Digital Converter (RDC) algorithm based on the arctangent [48]. To obtain the error, the position read with the EMFs of the resolver was compared to the position obtained with the ideal sine and cosine. Then, the maximum value of the error was considered.

Once the accuracy optimisation was carried out, the electrical design of the resolvers must be perforemd in order to calculate the power consumption of the resolver and the resolver’s total mass. For this purpose, a common input voltage, frequency, and Transformation Ratio (TR) were defined. The input voltage was 5 V, the frequency was 2.5 kHz, and the TR was 0.5, which is a common value among the commercial resolvers where different options with TRs of 0.454 and 0.5 can be found ([49,50,51]). Thus, the output voltage amplitude of each resolver was 2.5 V. Hence, the objective was to achieve a 2.5 V output while trying to minimize the power consumption of the resolver.

**Figure 3:**
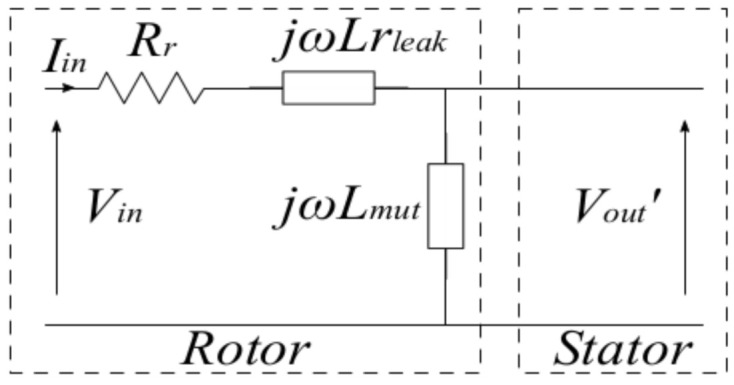
Equivalent circuit referring to the rotor.

Taking into account that the resolver operation principle is that of a transformer, the calculations were carried out based on the transformer’s equivalent circuit shown in Figure 3, where Rr is the rotor winding resistance, $L_{r_{leak}}$ is the leakage inductance, $L_{mut}$ is the mutual inductance, and $V_{in}$, $I_{in}$, and $V_{out}^{{}^{\prime }}$ are the input voltage, input current, and output voltage referred to the rotor, respectively. Moreover, as the resolver output is on an open circuit, the calculations are simplified as only the input parameters must be known. Then, the next steps were followed.

To calculate the inductances, Equations (1) and (2) were used.

(1) $$L_{r_{leak}}={\Lambda_{r}}_{{}_{leak}}\cdot {N_{r}}^{2}$$

(2) $$L_{mut}=\Lambda_{mut}\cdot {N_{r}}^{2}$$

where ${\Lambda_{r}}_{{}_{leak}}$, $\Lambda_{mut}$ and $N_{r}$ are the rotor leakage permeance, mutual permeance, and the turns of the rotor winding. The permeances were obtained from FEM simulation with Equations (3) and (4):

(3) $${\Lambda_{r}}_{{}_{leak}}=\frac{\Psi_{r_{sim}}-\Psi_{s_{sim}}}{{N_{r_{sim}}}^{2}\cdot I_{in_{sim}}}$$

(4) $$\Lambda_{mut}=\frac{\Psi_{s_{sim}}}{N_{r_{sim}}\cdot N_{s_{sim}}\cdot I_{in_{sim}}}$$

where $\Psi_{r_{sim}}$, $\Psi_{s_{sim}}$, $I_{in_{sim}}$, $N_{s_{sim}}$, and $N_{r_{sim}}$ are the linked flux by the rotor winding, the linked flux by the stator winding, the input current, the turns of the stator winding, and turns of the rotor winding used in simulation, respectively. Once the inductances are known, the other element to complete the circuit is the rotor winding’s resistance, which is calculated with Equation (5):

(5) $$R_{r}=\frac{\rho_{cu}\cdot l_{cu}}{S_{cu}}$$

where $\rho_{cu}$, $l_{cu}$, and $S_{cu}$ are the copper’s resistivity, length, and section, respectively. Once the resistance and inductances are known, the input impedance can be calculated. With this and the input voltage, the input current can be calculated. With the input voltage and current, the input power was obtained. With the current and the mutual impedance, the output voltage referring to the rotor was calculated. Finally, with Equation (6), the real output voltage at the stator was found.

(6) $$V_{out}={V_{out}}^{\prime }\cdot \frac{N_{s}}{N_{r}}$$

where Ns is the number of turns of the stator. With these equations, the number of turns of the stator and rotor could be varied in order to obtain the required voltage at the output mentioned previously while trying to while minimizing the input power.

Regarding the weight is influenced by two variables: the amount of ferromagnetic material needed and the amount of copper needed, as these are the two materials that will be used on the resolver construction. Regarding the ferromagnetic material, as said in Section 4, only the parameters shown in Figure 2 were optimized. In consequence, the teeth and yoke widths were not optimized, which are parameters that could affect the weight, and thus lighter resolvers could be achieved. In spite of this, the defined parameters were similar in each topology in order to make the comparison as fair as possible.

The outer diameter and length of each resolver were 18 mm and 10 mm, and the results obtained with each resolver are shown in the next sections and summarized in Table 2.

### 4.1. Slotted Full Pitch (4 Slots)

**Figure 4:**
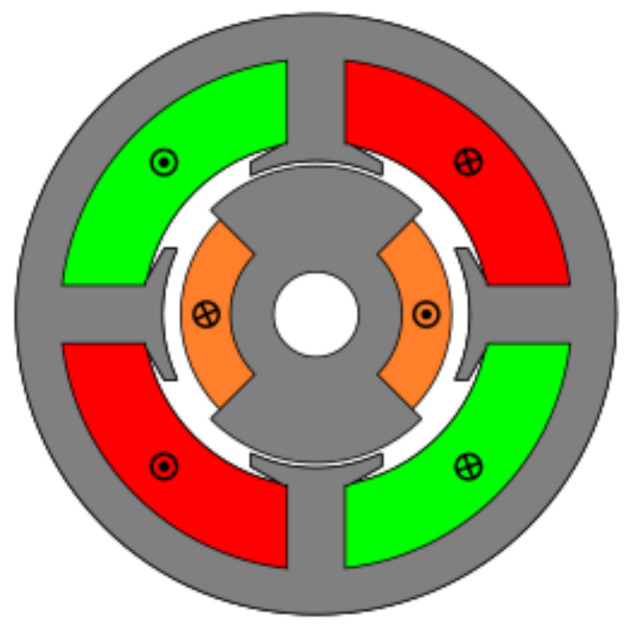
Full pitch winding slotted resolver with four slots.

The first studied topology was a resolver with four slots and full pitch winding as seen in Figure 4. For this topology, the tooth pitch and rotor pole pitch were optimized.

**Figure 5:**
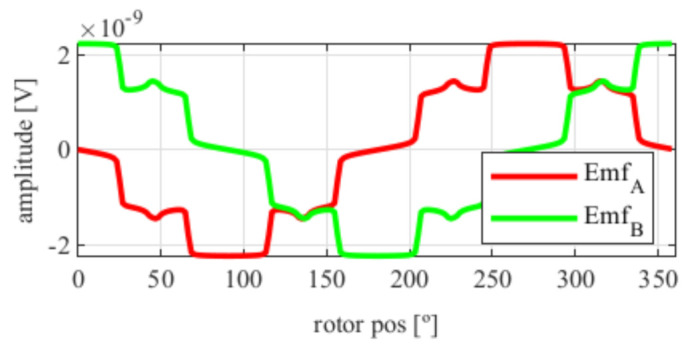
Full pitch four-slot resolver EMFs with the least THD.

As it can be seen in Figure 5, the EMFs are far from being perfectly sinusoidal as is desired, which means many harmonics exist. In order to improve this, skew was applied.

**Figure 6:**
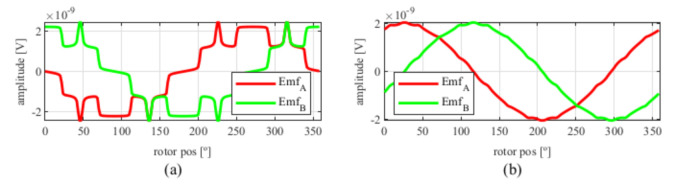
Full pitch four-slot resolver EMFs skewed for the least THD (**a**) before skew and (**b**) with skew.

After applying the skew, the results shown in Figure 6 were obtained. In Figure 6a, the original EMFs to which the skew was applied, and, in Figure 6b, the EMFs with skew are shown. Comparing Figure 6a to Figure 5, it can be deduced that the ks and kr parameters for the resolver with and without skew are different, thus, it is important that, for each topology the skew must be analysed for every parameter combination.

**Figure 7:**
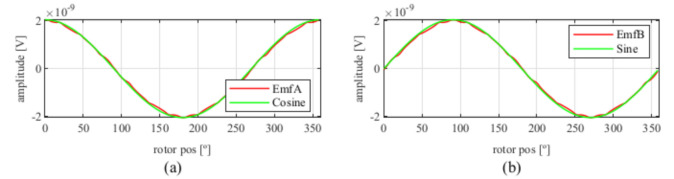
Full pitch four-slot skewed resolver EMFs vs. ideal signals (**a**) EMF A vs. Cosine and (**b**) EMF B vs. Sine.

**Figure 8:**
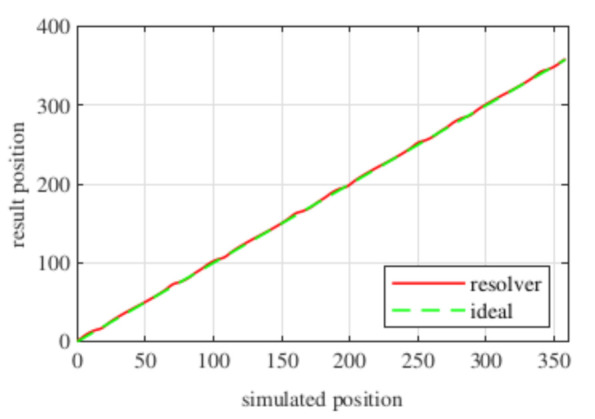
Full pitch four-slot skewed resolver position vs. the ideal position.

**Figure 9:**
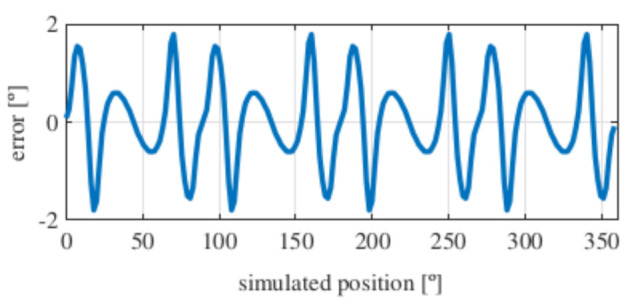
Full pitch four-slot skewed resolver position error.

Observing Figure 6b, most of the existent harmonic content was eliminated, obtaining more sinusoidal resolver signals. These were compared with the ideal sine and cosine waves in Figure 7. Then, these signals were converted to position Figure 8, and finally the error was calculated Figure 9).

As it can be seen, the error obtained with the full pitch four-slot resolver was around 1.8${}^{\circ }$ after applying the skew. The required power was 22 mW, and the calculated total mass was 38 g.

### 4.2. Slotted Short Pitch (4 Slots)

**Figure 10:**
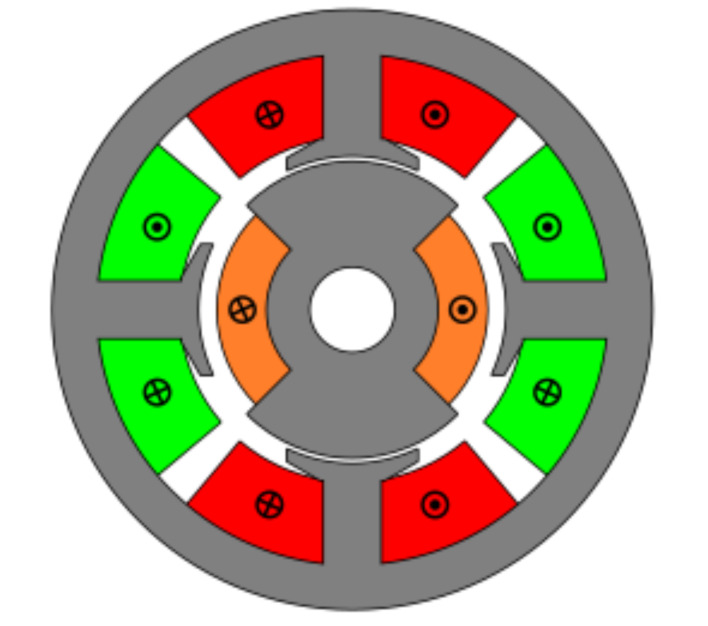
Short pitch winding slotted resolver with four slots.

The second studied topology was similar to the first one; however, in this case, the winding is short pitch as in Figure 10. As with the previous four-slot full pitch resolver, for this topology, the tooth pitch and rotor pole pitch were optimized.

**Figure 11:**
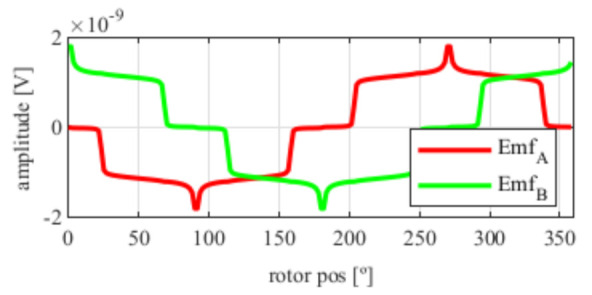
Short pitch four-slot resolver EMFs with the least THD.

**Figure 12:**
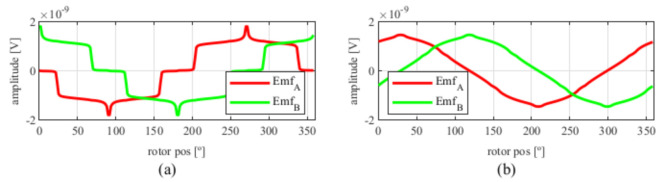
Short pitch four-slot resolver EMFs skewed for the least THD (**a**) before skew and (**b**) with skew.

As it can be seen in Figure 11, the EMFs were far from being perfectly sinusoidal. In order to improve this, different skew values were studied following the same strategy as with the previous topology. With skew, the results shown in Figure 12 were obtained.

**Figure 13:**
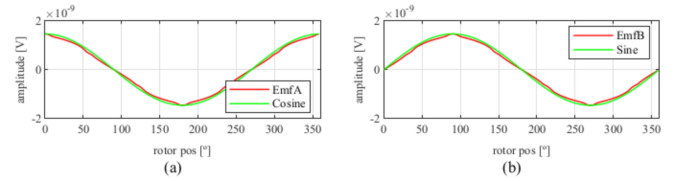
Full pitch four-slot skewed resolver EMFs vs ideal signals (**a**) EMF A vs. Cosine and (**b**) EMF B vs. Sine.

**Figure 14:**
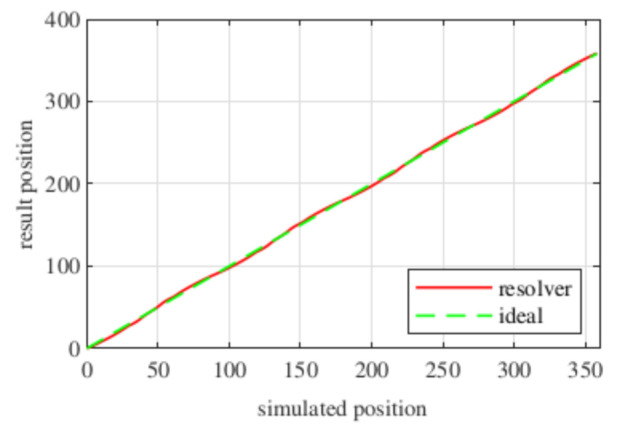
Short pitch four-slot skewed resolver position vs. the ideal position.

**Figure 15:**
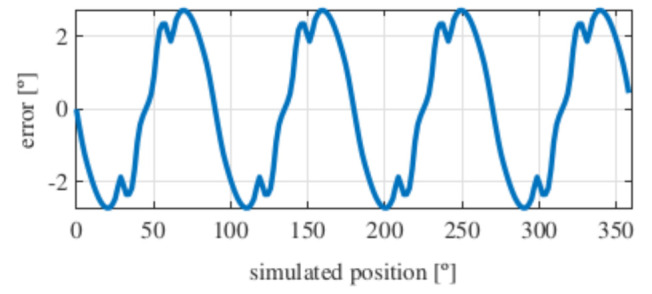
Short pitch four-slot skewed resolver position error.

With this resolver, the signals were improved as well, obtaining more sinusoidal signals (Figure 13). The position obtained with this resolver (Figure 14) was compared with the ideal position, and the error shown in Figure 15 was obtained.

With this topology, the obtained error was slightly more than 2${}^{\circ }$ after applying the skew, which was slightly higher than with the full pitch. The power consumption of this resolver was 17 mW, and its calculated mass was 38 g.

### 4.3. Slotted Toroidal Winding

**Figure 16:**
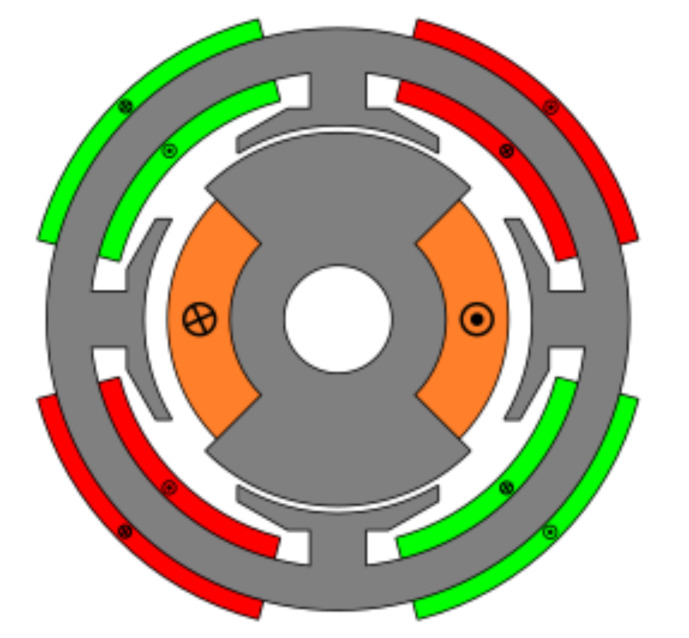
Toroidal winding slotted resolver with four slots.

The third studied topology had four slots as with the first two. In this case, in order to study another type of winding and find the best configuration, toroidal windings were added as shown in Figure 16. In this case, the optimized parameters were the rotor pole and stator tooth pitch as with the first two resolvers, keeping the stator coil pitch constant. This is due to the fact that, whichever the coil pitch, as the magnetic flux is gathered from the teeth, the same amount of flux will pass through each coil.

**Figure 17:**
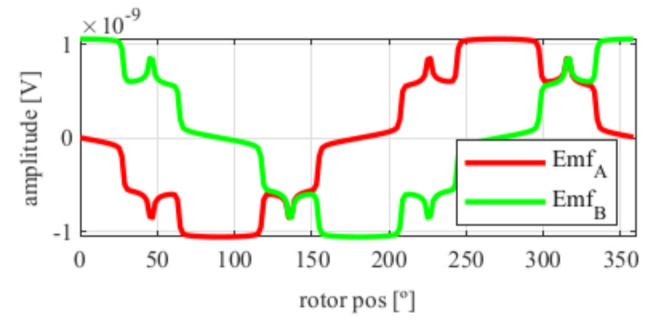
Toroidal four-slot resolver EMFs with the least THD.

**Figure 18:**
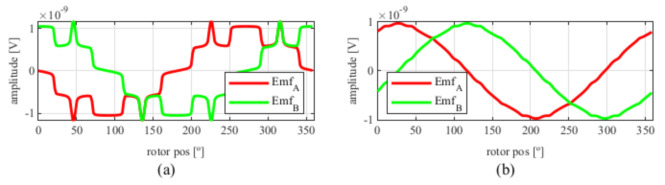
Toroidal four-slot resolver EMFs skewed for the least THD (**a**) before skew and (**b**) with skew.

From Figure 17, the EMFs were very similar to those obtained in Figure 5 with the four-slot full pitch resolver. Hence, following the steps of previous resolvers skew was applied, obtaining the results shown in Figure 18.

**Figure 19:**
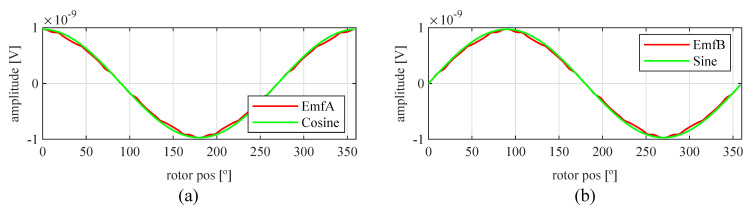
Toroidal four-slot skewed resolver EMFs vs. ideal signals (**a**) EMF A vs. Cosine and (**b**) EMF B vs. Sine.

**Figure 20:**
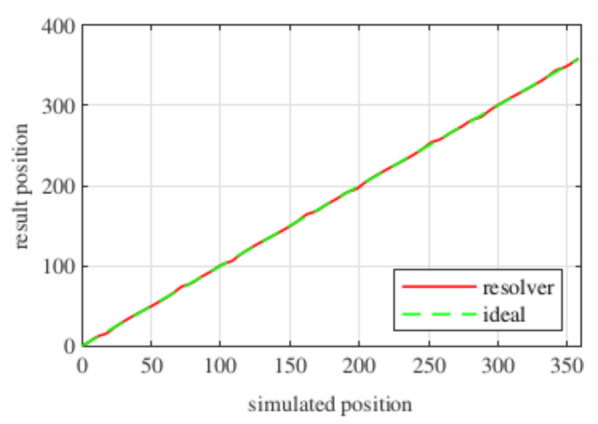
Toroidal four-slot skewed resolver position vs. the ideal position.

**Figure 21:**
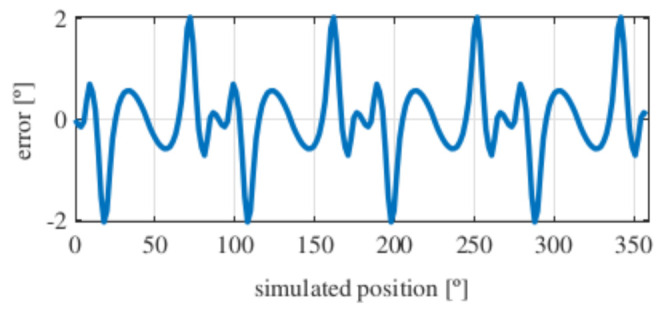
Toroidal four-slot skewed resolver position error.

Again, from Figure 18 and Figure 19, very similar results to those of the four-slot full pitch resolver shown in Figure 6 were obtained. However, in this case, after comparing the ideal and resolver’s position (Figure 20) and calculating the error in Figure 21, the error was higher than that in Figure 9.

With this resolver, the obtained error was 2${}^{\circ }$, being very similar to the slotted full pitch resolver. However, the performance was not improved, even though it has demonstrated to be better than the short pitch four-slot resolver. This resolver consumed 53 mW of power, and its total mass was 31 g.

### 4.4. Slotted Full Pitch (12 Slots)

**Figure 22:**
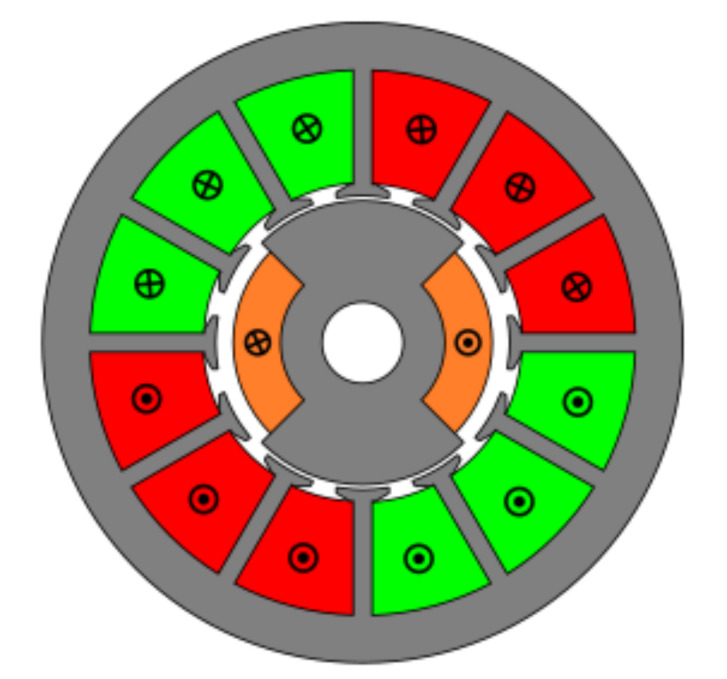
Full pitch winding slotted resolver with twelve slots.

The fourth studied topology had, again, a full pitch winding as was demonstrated to be the best winding for slotted structures. In this case, instead of having four slots, it had 12 as in Figure 22. The aim of increasing the number of slots was to reduce the lower frequency harmonic content and to consequently improve the obtained EMF signal quality.

**Figure 23:**
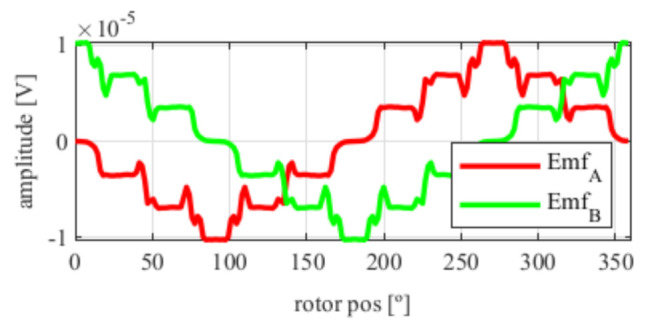
Full pitch twelve-slot resolver EMFs with the least THD.

**Figure 24:**
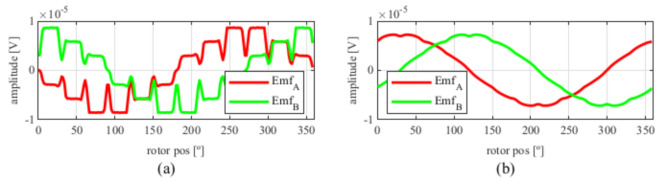
Full pitch twelve-slot resolver EMFs skewed for the least THD (**a**) before skew and (**b**) with skew.

As it can be seen in Figure 23, the EMFs were far from being perfectly sinusoidal. However, comparing them with the results in Figure 5, the most noticeable harmonics were of higher frequency. In order to improve this, skew was applied, thereby, obtaining the results in Figure 24.

**Figure 25:**
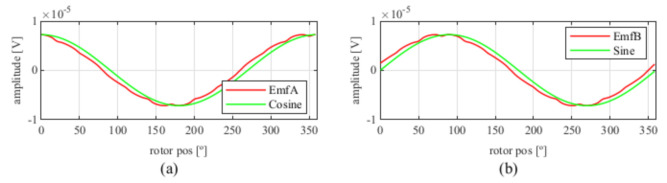
Full pitch twelve-slot skewed resolver EMFs vs. ideal signals (**a**) EMF A vs. Cosine and (**b**) EMF B vs. Sine.

**Figure 26:**
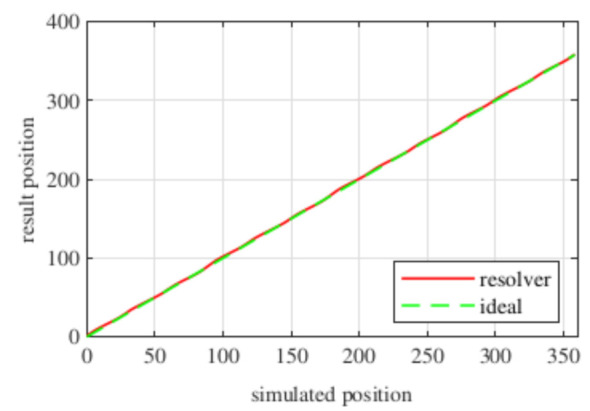
Full pitch twelve-slot skewed resolver position vs. the ideal position.

**Figure 27:**
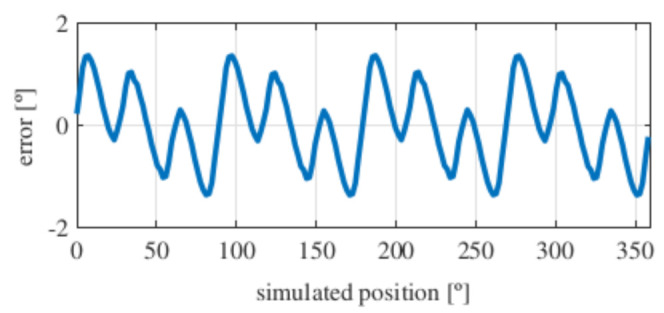
Full pitch twelve-slot skewed resolver position error.

With this resolver, the signals could be improved as well. As with the four-slot full pitch resolver, the geometry to obtain the EMFs with the lowest THD with and without skew were different. The obtained signals are compared with the ideal sine and cosine in Figure 25. The angular position obtained with this resolver (Figure 26) was compared with the ideal position. Then, the error shown in Figure 27 was calculated.

With this topology, the obtained error was lower than 1.5${}^{\circ }$, providing a slightly better accuracy than the four-slot full pitch resolver. The consumed power of this resolver was 22 mW, and the weight was 33 g.

### 4.5. Toroidal Winding (Slotless)

**Figure 28:**
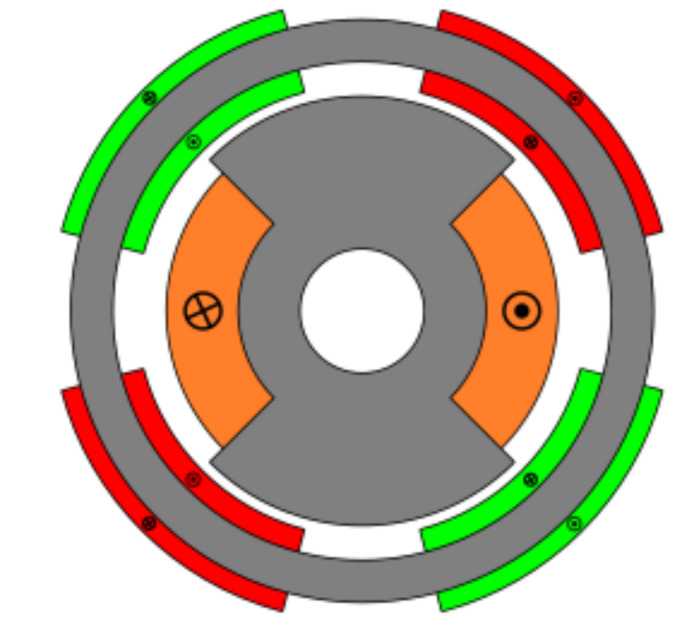
Toroidal winding slotless resolver.

The last studied resolver was a novel topology designed to improve the accuracy of absolute position resolvers. This novel topology had no slots and had a toroidal winding as shown in Figure 28. The key of this novel topology is that it has the same permeance around all the air gap, and thus EMFs with less harmonics; consequently, better quality should be expected. In this case, the optimized parameters were the rotor pole as in the previous topologies; however, in this case, the stator the coil pitch was optimized as there were no teeth (Figure 2b).

**Figure 29:**
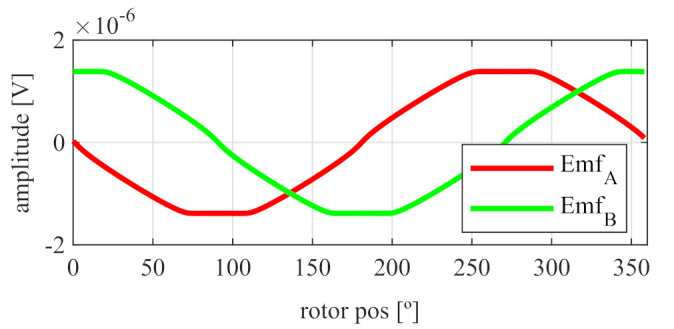
Toroidal slotless resolver EMFs with the least THD.

**Figure 30:**
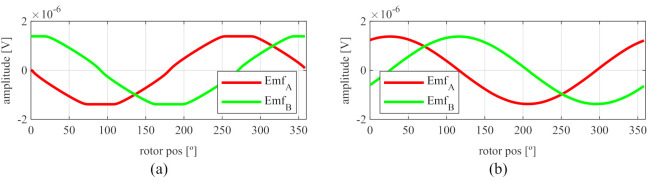
Toroidal slotless resolver EMFs skewed for the least THD (**a**) before skew and (**b**) with skew.

As can be seen in Figure 29, the signals obtained with the toroidal resolver were much more sinusoidal without skew than with the previous topologies. This was due to the airgap permeance, which was constant around all the gap as mentioned before. However, it can be seen that some noticeable harmonic content existed as the tips of the EMFs were flat. Following the procedure used with the other topologies, skew was applied, obtaining the results shown in Figure 30.

**Figure 31:**
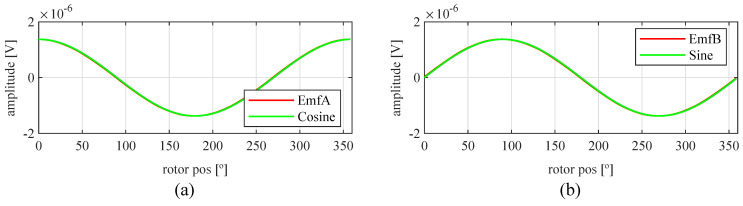
Toroidal slotless skewed resolver EMFs vs. ideal signals (**a**) EMF A vs. Cosine and (**b**) EMF B vs. Sine.

**Figure 32:**
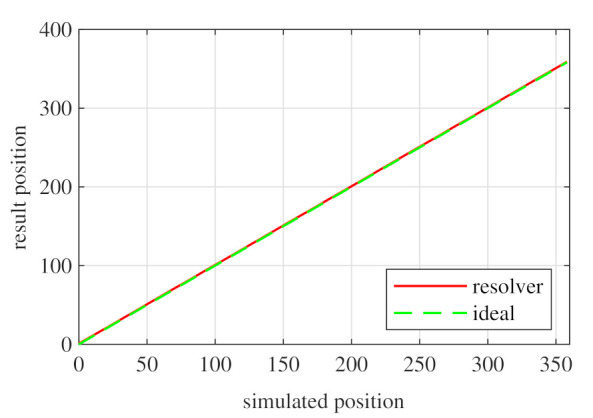
Toroidal slotless skewed resolver position vs. the ideal position.

**Figure 33:**
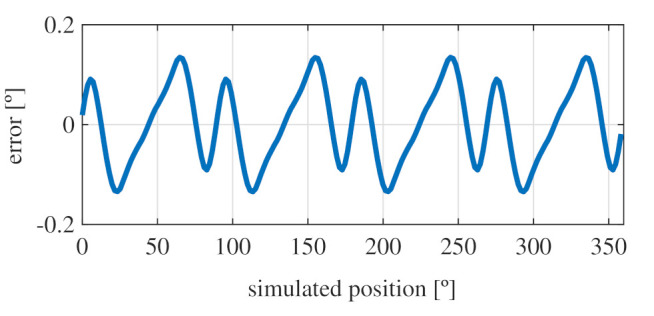
Toroidal slotless skewed resolver position error.

Comparing the skewed signals with the ideal sine and cosine, unlike the previous topologies, it can be seen that there were almost no observable differences (Figure 31). However, after comparing the position results (Figure 32) and calculating the error (Figure 33), the toroidal resolver was not perfect even though its performances weree much better than those demonstrated by other topologies. As it can be seen in Table 2, an error 10 times smaller was obtained.

With the slotless toroidal resolver, the obtained error was less than 0.15${}^{\circ }$, or in other words, a little more than than 8 arcminutes. The calculated power consumption for the novel resolver was 100 mW, and the weight was 33 g.

## 5. Results Discussion

In this section, the results obtained previously with each topology are discussed in order to find the most suitable topology for space applications. Taking into account the requirements explained in Section 1, having a high precision with an absolute position was desired as the robustness is intrinsic to the selected sensor.

In order to have a clearer perspective of the obtained results with each studied topology, the Table 2 was generated where the obtained best THD, the obtained maximum error, the consumed power, and each topology’s weight are summarized.

The studied maximum skew value was 60${}^{\circ }$ in order to not complicate the manufacturing and construction. After applying the skew to each topology, we observed that the THD decreased by more than ten times for each one, even reaching values below 2% for the slotted topologies and a 0.19% for the slotless.

In consequence, the THD improvement was reflected in the accuracy, which was around 2${}^{\circ }$ for the slotted ones and 0.135${}^{\circ }$ for the slotless. In terms of these values, the slotless toroidal topology was clearly the best topology as it provided the best results (more that ten-times better) than the others, even reaching an error of 0.135${}^{\circ }$ (8.1 arcminutes).

Regarding the slotted resolvers, the one that provided the least error was the 12-slot topology. As it has been said in Section 4.4, the addition of more slots was done aiming to decrease the lower frequency harmonic content, which could be the reason for obtaining better results. However, the obtained accuracy improvement may not be considered worthy assuming that six full pitch windings have to be mounted, consequently, increasing its construction difficulty. Hence, from the slotted topologies, the full pitch four-slot resolver is preferred.

However, in terms of power, the slotless resolver was demonstrated to be the one that consumed the most (up to five-times more than other topologies). This is due to the fact that the effective air-gap in this topology was much higher as there were no teeth, and the space required by the coils must be considered in the air-gap. Hence, in order to achieve the same TR as the other topologies, the slotless resolver consumed more power [52,53]. In this case, the topology that consumed the least was the short pitch four-slot resolver, even though the other topologies with full pitch winding were very close (only a 5 mW difference).

Regarding the weight, the full pitch 12-slot resolver was demonstrated to be the lightest. This could be in part due to the fact that toroidal winding takes advantage of all of the turns and has no head; hence, it does not have any "useless" weight. In case of the slotless, in addition to the toroidal winding, as it has no teeth, it could be thought that it should be lighter. However, as was mentioned previously, due to the required high air-gap, it needs more turns to achieve the defined output voltage, and hence it has more weight.

Additionally as this study is based on simulations, it was not possible to carry out a manufacturing cost analysis for each topology, even though this could be a key point in further studies. In spite of this, we found that, in the case of motors, the slotless topologies tended to be cheaper as they have a simpler structure [52,54].

The novel slotless topology proved to be the best, as the achieved accuracy was by far the best, and the increased power consumption was considered to be compensated.

Comparing the slotless resolver with the other technologies, the achieved accuracy was far from the 25 bits (0.04 arcseconds) offered by the optical encoder developed by Codechamp [11]. However, its high price due to the dominant position of the manufacturer makes it unsuitable for New Space, as lower budgets are sought. Additionally, the optical encoders are less suitable for harsh environments because of many reasons, including optical disc misalignment due to launch vibrations or due to dust or oil contamination. These would make the optical encoder useless, whereas the resolver is a proofed robust solution for harsh environments.

Moreover, comparing the novel resolver’s accuracy (8.1 arcminutes) with a commercial magnetic encoder of 1.5 arcminutes for space developed by Exxelia [55], they are both in the same measuring range, even though the encoder is better. In spite of this, the developed novel resolver could be a better alternative in some applications considering that the magnetic encoder is more sensible to external interferences, for instance, the electromagnetic fields created by a electric motor to which the encoder is attached, and hence it requires additional shielding [56].

Comparing the novel resolver with a potentiometer for space developed by Exxelia [57], in terms of accuracy, the potentiometer is much better (8.1 arcminutes of the resolver against 0.33 arcminutes (20 arcseconds) of the potentiometer). However, as has been explained in Section 2.1, the potentiometers have a dead zone where the position cannot be read. Due to this, a complementary encoder is required to provide position information in the entire revolution, whereas the accuracy is the same.

Regarding the resolver, the advantage of the additional space required by two potentiometers could be taken. This could be done by means of designing another multiple speed resolver. The combination of both resolvers would allow much better accuracies (in the range of arcseconds) while having an absolute position. Consequently, the resolver could be a better alternative.

Finally, even though commercially available resolvers for space with accuracies in the range of arcseconds can be found, they are multispeed, and hence they must be combined with a single speed resolver to provide an absolute position with those accuracies [49,50,51]. This could also be achieved by the novel resolver as explained in the previous paragraph. Moreover, even though there are some single speed resolvers with better accuracy, the novel developed resolver performed better or provided similar accuracies compared with most of the single speed resolvers.

Regarding the power, the novel topology consumed more than many of the commercially available options. As, in this study, the required assembly was not taken into account, we considered that the weight comparison could not be done. In spite of this, the calculated weight of the novel topology was lighter, and, as a consequence, its total mass with assembly could be similar to the existing solutions.

## 6. Conclusions

The sensors used for space applications are one of the key technologies with great potential to be improved by the introduction of new types in the New Space environment as described in the introduction. The resolvers are interesting solutions for space application because of their robustness and high accuracy.

The literature review of different revolver topologies showed that the reluctant alternatives are dependant on the fabrication accuracy. This makes a WR solution more attractive for mass production, which will be a key factor in New Space.

Different WR resolver topologies were designed and simulated in order to find the best one. In this study, a novel topology was designed—the toroidal winding slotless resolver. This new topology was proven to provide an absolute position with accuracies of around 8 arcminutes. This accuracy was higher than the accuracies found in the literature for the classical design of WR technology resolvers with single speed configuration. Additionally, the achieved technology’s accuracy improved or was very close to most of the commercially available single speed resolvers for aerospace. This higher accuracy is because the toroidal stator resolver output has lower harmonic distortion.

## Figures and Tables

**Table 1:** Resolver review summary.

Ref.	Topology	Type	Speed	Error (Simu.)	Error (Proto.)	Description
[27]	VR	Radial	2×	≤0.05${}^{\circ }$	0.614 ${}^{\circ }$	Fractional slot sinusoidal winding
[29]	VR	Radial	4×	≈15 arcmin	≈30 arcmin	Conventional
[31]	VR	Radial	2×	≈0.4${}^{\circ }$	≈0.4${}^{\circ }$	Two-pole distributed winding
[31]	VR	Radial	2×	≈5${}^{\circ }$	≈5${}^{\circ }$	Six-pole concentric winding
[33]	VR	Axial	2×	2.1 ${}^{\circ }$	≈2${}^{\circ }$	Disc type rotor
[33]	VR	Axial	4×	1.9 ${}^{\circ }$	≈2 ${}^{\circ }$	Disc type rotor
[33]	VR	Axial	8×	0.9 ${}^{\circ }$	≈1${}^{\circ }$	Disc type rotor
[35]	VR	Axial	1×	≈0.25${}^{\circ }$	≈0.25${}^{\circ }$	Disc type rotor
[36]	VR	Radial	4×	1.2 ${}^{\circ }$	2.6 ${}^{\circ }$	Slotless
[37]	VR	Radial	5×	0.08 ${}^{\circ }$	2${}^{\circ }$	Nonoverlapped winding
[38]	VR	Radial	5×	0.03 ${}^{\circ }$	1.4 ${}^{\circ }$	Alternate winding
[40]	VR	Radial	2×	≈1${}^{\circ }$	≈1.4${}^{\circ }$	Rotor with two ferromagnetic plates on a non-ferromagnetic shaft
[47]	VR	Radial	5×	≈0.25${}^{\circ }$	≈0.25${}^{\circ }$	Conventional
[47]	WR	Axial	3×	≈1.2${}^{\circ }$	≈1.2${}^{\circ }$	Conventional
[42]	WR	Axial	1×	1.1 ${}^{\circ }$	1.1 ${}^{\circ }$	Semi damper winding
[43]	WR	Axial	5×	0.3438 ${}^{\circ }$	0.3615 ${}^{\circ }$	Fractional slot concentrated winding
[44]	WR	Axial	10×	≈0.1${}^{\circ }$	≈0.2${}^{\circ }$	Fractional slot concentrated winding
[45]	WR	Axial	3×	4.5 ${}^{\circ }$	5.4 ${}^{\circ }$	RT integrated in the resolver

**Table 2:** Results obtained with the studied resolver topologies.

Topology	Skew THD [%]	Error [${}^{\circ }$]	Power [mW]	Weight [g]
Full pitch four-slot	2.1	1.79	22	38
Short pitch four-slot	4.51	2.71	17	38
Toroidal four-slot	2.54	2.02	53	28
Full pitch 12-slot	2.69	1.36	22	30
Toroidal slotless (Novel)	0.19	0.135	100	33

## Data Availability

Not applicable.
